
JOURNAL INFORMATION
==============================
NLM Title Abbreviation: ScientificWorldJournal
Journal ID: ScientificWorldJournal
NLM Journal ID: 101131163
Journal ID: TSWJ

The Scientific World Journal

ISSN: 2356-6140
EISSN: 1537-744X
Publisher: Wiley

ARTICLE INFORMATION
==============================
PMCID: PMC6009422
PMID: 29973792
DOI: 10.1100/tsw.2002.18
Article ID: 674538
Article version: 1
Subjects: Short Report

Functional Genomics with YBL036c

Berenfeld Ludmilla *
Sokolova Olga
Rim Sun Hee
Ringe Dagmar
Brandeis University, Waltham, MA, USA
*Ludmilla Berenfeld: berenfel@brandeis.edu
Publication date: 2002
Electronic publication date: 2002 Jan 5
Volume: 2
First page: 35
Last page: 35
Copyright: Copyright © 2002 Ludmilla Berenfeld et al.
Copyright year: 2002
License: This is an open access article distributed under the Creative Commons Attribution License, which permits unrestricted use, distribution, and reproduction in any medium, provided the original work is properly cited.
License URL: https://creativecommons.org/licenses/by/3.0/

==============================
Advances in Gene Technology: The Genome and Beyond –
Structural Biology for Medicine (Proceedings of the 2002 Miami
*Nature Biotechnology* Winter Symposium)
*TheScientificWorld* 2002, 2(S2), 35
ISSN 1532-2246; DOI 10.1100/tsw.2002.18

### FUNCTIONAL GENOMICS WITH YBL036c

Ludmilla Berenfeld, Olga Sokolova, Sun Hee Rim and Dagmar Ringe
Brandeis University, Waltham, MA
berenfel@brandeis.edu

Functions of more than 2,000 genes in the *Saccharomyces cerevisiae* genome are still unknown. Gene YBL036c is among them. Gene YBL036c encodes a protein that is PLP (pyridoxal-phosphate) dependent. Its sequence does not indicate similarity to any known protein. However, its 3D structure, including the active site residues, are almost identical to that of bacterial alanine racemase. From the structure we can deduce that it is a racemase. An increasing number of data have shown the existence of D enantiomers of amino acids in eukaryotes. D,L-dityrosine was found in spore walls of *S. cerevisiae*[1] D-serine - in mammalian brain cells[2], and an accumulation of some amino-acids in D-form in human tissue, such as bone and eye lens, has been described[3]. Therefore, it seems very interesting and important to recognize a possible racemase activity, which is required for existence and/or amino acid metabolism in eukaryotes.

Using YBL036c deleted and isogenic parental S288c strains, we have discovered the phenotype of the deleted gene. We have observed a significant morphological change in the spore tetrad envelope structure. During prolonged sporulation, theYBL036c deleted strain produces sticky fibrous elements, visible by the naked eye in a liquid culture. The spores appear as a difficult-to-separate, highly adhesive mass of sticky spores. This morphological abnormality is shown by electron microscopy as filament-like structures on the surface of tetrad envelopes. We found that this effect increases during further incubation of the spore culture.

Our preliminary microarray data of the comparison between YBL036 deleted and parental strains, have demonstrated the change in the expression pattern of more than 200 genes. Many of these genes are involved in the cell wall biosynthesis pathway. That is consistent with the observed phenotype. By analyzing the activity of purified protein and its crystal structure, we expect to determine the specific functional activity of the YBL036c encoded protein. This work is currently in progress.

### REFERENCES

1. Briza, P., Ellinger, A., Winkler, G., and Breitenbach, M. (1990) J. Biol. Chem. 265, 15118–15123.
2. Soutourina, J., Plateau, P., and Blanquet, S. (2000) J. Biol. Chem. 275, 32535–35542.
3. Uo, T., Yoshimura, T., Tanaka, N., Takegawa, K., and Esaki, N. (2001) J. Bacteriol. 183, 2226–2233.
